# Epistatic Roles for *Pseudomonas aeruginosa* MutS and DinB (DNA Pol IV) in Coping with Reactive Oxygen Species-Induced DNA Damage

**DOI:** 10.1371/journal.pone.0018824

**Published:** 2011-04-18

**Authors:** Laurie H. Sanders, Babho Devadoss, Geraldine V. Raja, Jaime O'Connor, Shengchang Su, Daniel J. Wozniak, Daniel J. Hassett, Anthony J. Berdis, Mark D. Sutton

**Affiliations:** 1 Department of Biochemistry, School of Medicine and Biomedical Sciences, University at Buffalo, State University of New York, Buffalo, New York, United States of America; 2 Witebsky Center for Microbial Pathogenesis and Immunology, School of Medicine and Biomedical Sciences, University at Buffalo, State University of New York, Buffalo, New York, United States of America; 3 Department of Pharmacology, Case Western Reserve University, Cleveland, Ohio, United States of America; 4 Department of Molecular Genetics, Biochemistry, and Microbiology, University of Cincinnati, Cincinnati, Ohio, United States of America; 5 Department of Infectious Disease and Microbiology, Center for Microbial Interface Biology, The Ohio State University, Columbus, Ohio, United States of America; Louisiana State University and A & M College, United States of America

## Abstract

*Pseudomonas aeruginosa* is especially adept at colonizing the airways of individuals afflicted with the autosomal recessive disease cystic fibrosis (CF). CF patients suffer from chronic airway inflammation, which contributes to lung deterioration. Once established in the airways, *P. aeruginosa* continuously adapts to the changing environment, in part through acquisition of beneficial mutations *via* a process termed pathoadaptation. MutS and DinB are proposed to play opposing roles in *P. aeruginosa* pathoadaptation: MutS acts in replication-coupled mismatch repair, which acts to limit spontaneous mutations; in contrast, DinB (DNA polymerase IV) catalyzes error-prone bypass of DNA lesions, contributing to mutations. As part of an ongoing effort to understand mechanisms underlying *P. aeruginosa* pathoadaptation, we characterized hydrogen peroxide (H_2_O_2_)-induced phenotypes of isogenic *P. aeruginosa* strains bearing different combinations of *mutS* and *dinB* alleles. Our results demonstrate an unexpected epistatic relationship between *mutS* and *dinB* with respect to H_2_O_2_-induced cell killing involving error-prone repair and/or tolerance of oxidized DNA lesions. In striking contrast to these error-prone roles, both MutS and DinB played largely accurate roles in coping with DNA lesions induced by ultraviolet light, mitomycin C, or 4-nitroquinilone 1-oxide. Models discussing roles for MutS and DinB functionality in DNA damage-induced mutagenesis, particularly during CF airway colonization and subsequent *P. aeruginosa* pathoadaptation are discussed.

## Introduction

Despite the fact that most organisms are equipped with numerous DNA repair functions, DNA lesions often evade repair. If left unchecked, these lesions can block ongoing replication, leading to mutations, genome rearrangements, and even cell death [1]. One evolutionarily conserved mechanism by which bacteria tolerate replication blocking DNA lesions involves their direct bypass *via* a process termed translesion DNA synthesis (TLS) [1], [2]. Most if not all organisms possess multiple DNA polymerases (Pols) capable of catalyzing TLS, several of which belong to the Y-family [1], [3]. In general, members of this family of Pols possess a preformed and open catalytic active site compared to well studied high fidelity replicative Pols, are distributive, and lack intrinsic exonuclease proofreading activity (reviewed in [1], [4]). Taken together, these features confer upon Y-family Pols a reduced fidelity relative to most well studied replicative enzymes. This reduced fidelity is vital to their ability to catalyze TLS, and, together with the miscoding or non-coding nature of many lesions, explains why TLS can be error-prone, contributing to mutations.

The Y-family of Pols is comprised of four main subgroups, or branches (reviewed in [3]). The bacterial DinB (Pol IV; hereafter referred to as DinB)/eukaryotic Pol κ branch is the most evolutionarily conserved [3], [5], suggesting that its members play one or more vitally important roles with respect to DNA repair/damage tolerance. Although it is unclear whether the members of the DinB branch act in one or more conserved role, several distinct activities have been described for representative members. For example, both *E. coli* DinB and mammalian Pol κ are capable of catalyzing accurate bypass of *N^2^*-dG–furfuryl adducts [6], [7], as well as model *N^2^*-dG–*N^2^*-dG interstrand DNA cross-links [8], [9]. In addition, *E. coli* DinB plays an active role in contributing to mutations under conditions of limiting carbon source *via* an error-prone dsDNA break repair pathway [10], and may play a role in error-free bypass of cytotoxic alkylating DNA lesions [11]. Although *E. coli* DinB cannot catalyze bypass of UV photoproducts [12], the *Sulfolobus solfataricus* DinB ortholog, P2 Pol IV (Dpo4) can bypass a model *cis-syn* thymine cyclobutane dimer *in vitro* [13]. Human Pol κ is unable to bypass a model thymine dimer *in vitro*; however, it can extend a primer bearing a 3′-dG located opposite the 3′-dT of a model thymine cyclobutane dimer *in vitro* [14]. Moreover, Pol κ catalyzes error-prone bypass of 8-oxo-7,8-dihydro-2′-deoxyguanosine 5′-monophosphate (8-oxo-dG), and largely accurate bypass of 5,6-dihydro-5,6-dihydroxythymidine (thymine glycol) *in vitro* [15], [16], [17]. Finally, both *E. coli* DinB and human Pol κ incorporate 2-hydroxy-dATP opposite template-dG or -dT, as well as 8-oxo-dG opposite template-dA [18], [19]. Taken together, these findings illustrate the difficulty in predicting *a priori* the ability of a particular DinB/Pol κ enzyme to tolerate a specific lesion, and/or the fidelity with which a particular DinB/Pol κ enzyme will behave.

We recently initiated a study of the *Pseudomonas aeruginosa* DinB protein as part of a larger effort aimed at understanding mechanisms contributing to mutagenesis and adaptation [20]. *P. aeruginosa* is a human opportunistic pathogen that is commonly associated with a variety of human diseases, particularly chronic respiratory infections of cystic fibrosis (CF) patients (reviewed in [21], [22]). Following airway colonization, *P. aeruginosa* acquires mutations that confer an adaptive advantage, enabling the pathogen to persist within CF airways for years to decades, ultimately leading to the death of the patient [23], [24], [25], [26], [27], [28], [29], [30]. The process by which *P. aeruginosa* acquires adaptive mutations is referred to as ‘pathoadaptation.’ Mutational inactivation of the *P. aeruginosa mucA* gene results in a mucoid phenotype, and is one of the best-studied examples of an adaptive mutation directly correlated with persistent infections and poor clinical prognosis (reviewed in [31]). Based on two separate studies [32], [33], more than 80% of the mucoid *P. aeruginosa* strains isolated from individuals afflicted with CF were found to contain a mutation within *mucA*, suggesting that mutations inactivating this locus confer an advantage in airway pathogenesis. The *mucA* gene encodes an anti-sigma factor (MucA protein) that negatively regulates the actions of the alternative sigma factor, AlgT (or AlgU) (reviewed in [23], [31]). Inactivation of *mucA* leads to loss of MucA-mediated antagonism of AlgT, which in turn activates transcription of genes required for alginate production. A majority of *mucA* alleles impaired for regulation of AlgT contain a –1 frameshift mutation within a single homopolymeric run of 5 consecutive dG residues, referred to as the *mucA22* allele [32]. Our finding that *P. aeruginosa* DinB favored –1 frameshift mutations within poly-dA and poly-dG runs over GC→TA transversions nearly 3-to-1 led us to suggest that this Pol may contribute to *mucA* inactivation during airway infection [20]. Consistent with this hypothesis, DinB contributes to mutations in *mucA* that promote alginate production under laboratory conditions [34]. This same study revealed that mutational inactivation of MutS, which together with MutL and UvrD enables mismatch repair (MMR), served to significantly increase the frequency of *mucA* mutations.

MMR is a replication-coupled repair function that acts to correct replication errors, thereby limiting the frequency of spontaneous mutations. MMR function relies at a minimum on MutS, MutL, and UvrD. Based on seminal work in *E. coli*, MutS acts to recognize DNA mismatches (reviewed in [35]). After binding the mismatch, MutS recruits MutL. In some organisms, MutL possesses a nuclease activity that cleaves the daughter strand containing the replication error [36]. *P. aeruginosa* MutL has not yet been demonstrated to possesses nuclease activity. MutL also acts to recruit UvrD, a DNA helicase (helicase II) that loads at the nick site to unwind the duplex DNA, leaving a single strand (ss) DNA gap. This ssDNA gap is subsequently filled in by action of the replicative DNA polymerase, and the resulting nick sealed by DNA ligase. Loss-of-function mutations in either *mutS*, *mutL*, or *uvrD* abolish MMR, leading to significantly elevated spontaneous mutation frequencies, due in large part to an inability to correct replication errors [35]. More recently, roles for MutS, MutL, and UvrD in addition to MMR have been suggested. For example, MutS may participate in base excision repair, MutL was recently determined to interact with a variety of proteins that participate in functions other than MMR, and UvrD participates in nucleotide excision repair, and also plays an antagonistic role in recombination by dismantling RecA/ssDNA nucleoprotein filaments [37], [38], [39], [40], [41]. Taken together, these findings suggest that DNA lesions in addition to mismatched base pairs might contribute to the elevated mutation rates observed for MMR-deficient strains. Finally, of significance to work discussed in this report, a significant fraction of CF patients harbor a population of *P. aeruginosa* that displays an elevated spontaneous mutation frequency (*e.g*., hypermutable phenotype) due to loss of MMR function, most frequently resulting from mutational inactivation of *mutS* [24], [25], [28], [42].

Despite the fact that a large fraction of CF patients are colonized by *mutS*-deficient *P. aeruginosa* strains [24], [25], [28], [42], there is a paucity of information regarding the biology of these strains. Based on a murine airway infection model, inactivation of *mutS* renders *P. aeruginosa* less virulent [28], [43]. *P. aeruginosa* is exposed to high levels of reactive oxygen species (ROS) while colonizing CF airways, especially in the early “aerobic” phases (reviewed in [21], [22]). As a result, *P. aeruginosa* experiences significant levels of DNA damage. Inasmuch as MutS acts to prevent mutations, we hypothesized that MutS function might contribute to accurate repair of ROS-damaged DNA, and that in the absence of MutS, *P. aeruginosa* would be impaired for coping with these lesions, possibly explaining the reduced virulence of this strain in a murine model [28], [43]. Moreover, since oxidized DNA lesions can be tolerated by TLS, we further hypothesized an important role for DinB in protecting *P. aeruginosa* against ROS-induced DNA damage. Finally, since error-prone repair or TLS of oxidized DNA lesions will contribute to mutations, we hypothesized that MutS and DinB play opposing roles in *P. aeruginosa* pathoadaptation. While testing these hypotheses using isogenic *mutS*- and/or *dinB*-deficient strains, we unraveled an unexpected epistatic relationship between *mutS* and *dinB* functionality with respect to H_2_O_2_-induced cell killing. Our discovery that both *dinB* and *mutS* functionality contributed to H_2_O_2_-induced mutagenesis suggests that H_2_O_2_ sensitivity of the *mutS* and *dinB* strains was the direct result of their impaired ability to cope with ROS-induced DNA damage. In striking contrast to these error-prone roles, both MutS and DinB played accurate roles in tolerating and/or repairing DNA lesions induced by ultraviolet light (UV), mitomycin C (MMC), or 4-nitroquinilone 1-oxide (4-NQO) exposure. Models discussing roles for MutS and DinB functionality in DNA lesion repair and/or tolerance, as well as DNA damage-induced mutagenesis, particularly during CF airway colonization and subsequent *P. aeruginosa* pathoadaptation are discussed.

## Results

### Contributions of *P. aeruginosa* DinB and MutS to spontaneous mutagenesis

**Figure 1:**
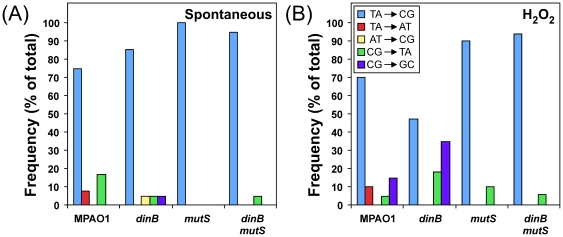
Summary of spontaneous and H_2_O_2_-induced base substitutions in *rpoB* that confer Rif^R^. Results of *rpoB* DNA sequence analysis for spontaneous (**A**) and H_2_O_2_-induced (**B**) Rif^R^ *P. aeruginosa* mutants are summarized with respect to the types of nucleotide substitution observed. Frequency refers to the occurrence of each observed base substitution mutation as a function of the total number of spontaneous or H_2_O_2_-induced Rif^R^ mutants sequenced for each strain. See Table 2 for details concerning the number of Rif^R^ clones analyzed for each strain, as well as the specific nucleotide position and substitution of each documented mutation.

As part of an ongoing effort to understand mechanisms contributing to *P. aeruginosa* pathoadaptation, we constructed a set of isogenic PAO1-derived strains bearing different combinations of wild-type and mutant *mutS* and *dinB* alleles. Prior to determining roles for *dinB* and/or *mutS* function(s) in DNA damage-induced mutagenesis, we measured their respective contributions to spontaneous mutagenesis. Inactivation of *mutS* (*mutS*::IS*phoA*/hah) conferred a ∼650-fold increase in the frequency of spontaneous Rif^R^ compared to the isogenic *mutS^+^* strain (Table 1), consistent with its well-established role in replication-coupled MMR [35]. Hypermutability of the *mutS* strain was fully complemented by plasmid p*mutS* (Table 1), which expresses the *P. aeruginosa* MutS protein from its native promoter. In contrast to *mutS*, deletion of *dinB* (Δ*dinB*::*aacC1*) failed to significantly influence the frequency of spontaneous Rif^R^, irrespective of *mutS* activity (Table 1). Based on nucleotide sequence analysis of representative spontaneously arising Rif^R^ clones for each of the four strains, mutations resulted exclusively from spontaneous base substitutions (Table 2), as expected given the essential role of the *rpoB* gene product (β subunit of RNA Pol) [44]. The overwhelming majority of observed mutations were TA→CG transitions, irrespective of *mutS* and *dinB* functionality (see Fig. 1A & Table 2). Importantly, GC→TA transversions, which are characteristic of DinB [20], were not observed. Taken together, these results indicate that DinB function does not contribute to spontaneous base substitutions in *P. aeruginosa*. In addition, they confirm a pivotal role for MutS in limiting spontaneous mutations.

**Table 1:** Contribution of *P. aeruginosa mutS* and *dinB* functionality to spontaneous Rif^R^.

Strain	Phenotype with respect to functionality of:^a^		Frequency of spontaneous Rif^R^ b	Fold-effect^c^
	MutS	DinB		
MPAO1	+	+	2.5 (1.4–3.0)×10^−8^	≡1.0
WFPA334	+	−	4.0 (1.6–4.0)×10^−8^	1.6
MPA32417	−	+	16.2 (12.9–19.0)×10^−6^	648
MPA3241 (p*mutS*)^d^	+^d^	+	2.1 (0.7–5.2)×10^-8^	0.8
UBPA100	−	−	15.1 (13.8–19.9)×10^−6^	604

***^a^***Relevant phenotypes with respect to *dinB* and *mutS* function are indicated: +, wild-type; −, loss-of-function.

***^b^***Median mutation frequency is shown. Calculated frequencies are based on either 5 (for strain MPA32417 bearing plasmid p*mutS*) or 30 (for all other strains) independent determinations. Values in parentheses represent 95% confidence intervals, and were calculated as described [68].

***^c^***Fold-increase in median spontaneous mutation frequency relative to MPAO1, whose frequency was set equal to 1.0 (≡1.0).

***^d^***Plasmid p*mutS* expresses the wild-type MutS protein (*mutS^+^*) from its native promoter.

**Table 2:** Nucleotide sequence analysis of *rpoB* alleles recovered from spontaneous or H_2_O_2_-induced Rif^R^ *P. aeruginosa* strains.^a^

Spontaneous *rpoB* mutations					
Nucleotide substitution^b^	Deduced amino acid substitution	Rif^R^ *P. aeruginosa* strains^c^			
		MPAO1 (WT)^d^	WFPA334 (*dinB*)	MPA32417 (*mutS*)	UBPA100 (*mutS dinB*)
CTG→CCG	L516→P	*nd*	*nd*	*nd*	1
TCG→CCG	S517→P	1	*nd*	*nd*	1
CAG→CGG	Q518→R	4	*nd*	*nd*	4
GAC→AAC	D521→N	1	*nd*	*nd*	1
GAC→GGC	D521→G	3	14	15	12
GAC→GTC	D521→V	1	*nd*	*nd*	*nd*
GAC→GCC	D521→A	*nd*	1^f^	*nd*	*nd*
CAG→GAG	Q522→E	*nd*	1^f^	*nd*	*nd*
AAC→GAC	N523→D	*nd*	1	*nd*	*nd*
CAC→TAC	H531→Y	*nd*	1	*nd*	*nd*
CAC→CGC	H531→R	*nd*	1	3	1
TCC→TTC	S536→F	1	*nd*	*nd*	*nd*
CTC→CCC	L538→P	1	*nd*	*nd*	*nd*

H_2_O_2_-induced *rpoB* mutations					
Nucleotide substitution^b^	Deduced amino acid substitution	Rif^R^ *P. aeruginosa* strains^c^			
		MPAO1 (WT)	WFPA334 (*dinB*)^g^	MPA32417 (*mutS*)	UBPA100 (*mutS dinB*)^h^
CTG→CCG	L516→P	*nd*	*nd*	1	*nd*
TCG→CCG	S517→P	*nd*	*nd*	1	*nd*
TCG→TTG	S517→L	*nd*	1	*nd*	1
CAG→CGG	Q518→R	*nd*	*nd*	1	*nd*
GAC→AAC	D521→N	*nd*	*nd*	2	*nd*
GAC→GGC	D521→G	10	8	15	17
CAC→GAC	H531→D	3	6	*nd*	*nd*
CAC→TAC	H531→Y	*nd*	2	*nd*	*nd*
CAC→CTC	H531→L	2	*nd*	*nd*	*nd*
CTC→CCC	L538→P	4	*nd*	*nd*	*nd*
CCT→CTT	P567→L	1	*nd*	*nd*	*nd*

***^a^***Spontaneous and H_2_O_2_-induced (25 mM H_2_O_2_) mutations were identified as described in *Materials and Methods*. The region of *rpoB* encompassing amino acids 499–582 of the β subunit of RNA polymerase was PCR amplified from 18–20 independent Rif^R^ clones for each strain and subjected to automated nucleotide sequence analysis.

***^b^***Each nucleotide substitution (underlined) is shown in the context of its respective codon.

***^c^***The number of times that each mutation was identified in the group of 18–20 that was sequenced is indicated; *nd*, the indicated mutation was not detected.

***^d^***Seven of the 19 Rif^R^ clones examined did not contain a mutation within amino acids 499–582.

***^e^***One of the 19 Rif^R^ clones examined did not contain a mutation within amino acids 499–582.

***^f^***These mutations were present in the same *rpoB* allele.

***^g^***Three of the 20 Rif^R^ clones examined did not contain a mutation within amino acids 499–582.

***^h^***Two of the 20 Rif^R^ clones examined did not contain a mutation within amino acids 499–582.

### DinB and MutS act epistatically to protect *P. aeruginosa* against H_2_O_2_-mediated killing

**Figure 2:**
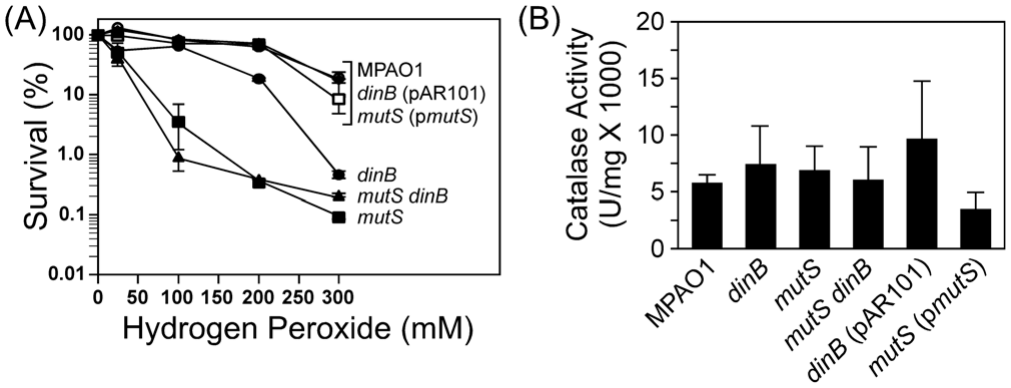
Contributions of *dinB* and/or *mutS* function to survival following exposure to ROS. Respective H_2_O_2_ sensitivities (**A**), and catalase activities for cell free extracts (**B**) of isogenic *P. aeruginosa* strains MPAO1 (wild-type) bearing pUCP20T, WFPA334 (Δ*dinB*::*aacC1*) bearing pUCP20T (control) or pAR101 (*dinB^+^*), MPA32417 (*mutS*::IS*phoA*/hah) bearing pUCP20T (control) or p*mutS* (*mutS^+^*), and UBPA100 (Δ*dinB*::*aacC1 mutS*::IS*phoA*/hah) bearing pUCP20T were determined as described in *Materials and Methods*. H_2_O_2_ sensitivities represent the average of 4 independent experiments, while catalase activities represent the average of 3 independent experiments; error bars represent the standard deviation. Based on a two-way ANOVA with Bonferroni post-test, there was a significant interaction between strain and concentration of H_2_O_2_ in (**A**), and differences in H_2_O_2_ sensitivity of wild-type and *mutS* and *dinB* strains were statistically significant (*p*<0.05). Based on a one-way ANOVA with Dunnett's post-test, differences in catalase activity in (**B**) were not significant (*p*>0.05).

The *dinB* and *mutS* genes are among the numerous *P. aeruginosa* genes whose transcription is induced following exposure to H_2_O_2_ [45], [46], suggesting roles for DinB and MutS in coping with ROS-induced DNA lesions. To test this hypothesis, we asked whether loss of *dinB* and/or *mutS* function(s) enhanced sensitivity of *P. aeruginosa* to H_2_O_2_. As summarized in Fig. 2A, wild-type *P. aeruginosa* (MPAO1) was relatively insensitive to killing by H_2_O_2_ over the concentration range examined. In contrast to MPAO1, the *mutS*-deficient strain (MPA32417) displayed a pronounced hypersensitivity to all H_2_O_2_ concentrations examined (Fig. 2A), suggesting an important role for MutS in sensing and/or repairing oxidized DNA lesions. H_2_O_2_ hypersensitivity was statistically significant (*p*<0.05, based on two-way ANOVA with Bonferroni post-test), and was fully complemented by the MutS-expressing plasmid (p*mutS*). The *dinB*-deficient strain (WFPA334) also displayed increased sensitivity to H_2_O_2_, although this sensitivity was largely restricted to higher levels of H_2_O_2_ (Fig. 2A), suggesting an important role for DinB in tolerating H_2_O_2_-induced DNA lesions that persist in the DNA, rather than their repair. Sensitivity of the *dinB* strain was statistically significant at concentrations ≥200 nM H_2_O_2_ (*p*<0.05), and was fully complemented by the DinB-expressing plasmid, pAR101. Finally, the *mutS dinB* double mutant strain (UBPA100) displayed a level of H_2_O_2_ sensitivity that was comparable to the *mutS*-deficient *dinB^+^* strain (Fig. 2A), indicating an epistatic relationship between *dinB* and *mutS* function(s) with respect to H_2_O_2_-mediated killing. As a control, we measured levels of catalase activity in cell-free extracts prepared from each strain. As summarized in Fig. 2B, each strain harbored comparable levels of catalase activity (*p*>0.05, based on one-way ANOVA with Dunnett's post-test), indicating that H_2_O_2_ sensitivity of the *mutS* and *dinB* strains was not the result of reduced catalase levels. These results, taken together with the known roles for DinB and MutS discussed above, support a model in which *P. aeruginosa* MutS and DinB act in a common pathway focused on coping with H_2_O_2_-induced DNA damage.

### Both DinB and MutS contribute to H_2_O_2_-induced mutagenesis in *P. aeruginosa*

**Figure 3:**
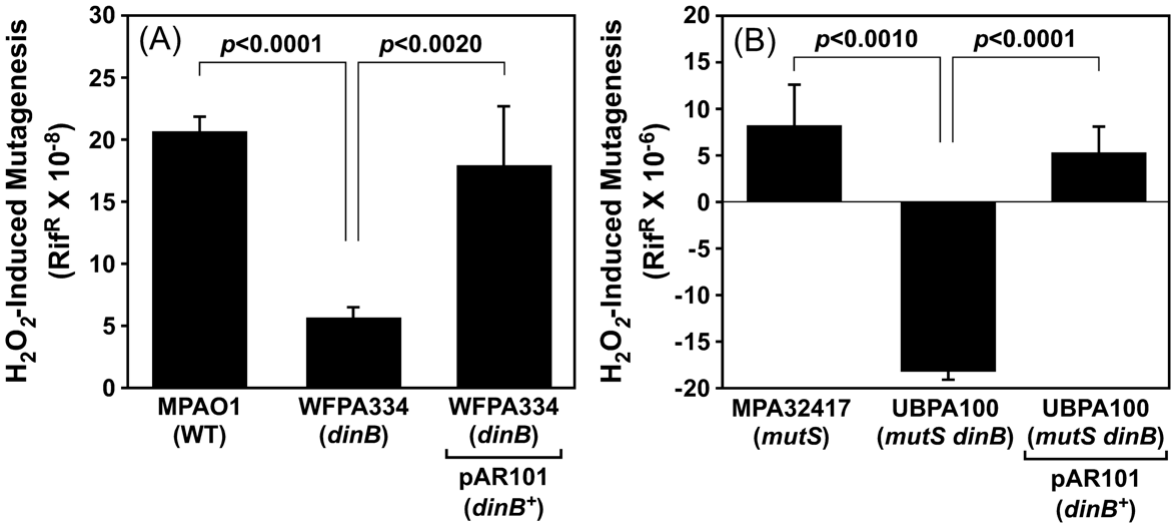
Contribution of *dinB* and/or *mutS* function to H_2_O_2_-induced mutagenesis. H_2_O_2_-induced mutagenesis was measured in *mutS^+^* (**A**), or *mutS*::IS*phoA*/hah (**B**) *dinB^+^* and Δ*dinB*::*aacC1* strains, bearing the indicated plasmids. Strains examined include MPAO1 (wild-type) bearing pUCP20T, WFPA334 (Δ*dinB*::*aacC1*) bearing pUCP20T (control) or pAR101 (*dinB^+^*), MPA32417 (*mutS*::IS*phoA*/hah) bearing pUCP20T (control), or UBPA100 bearing pUCP20T (control) or pAR101 (*dinB^+^*). H_2_O_2_-induced mutation frequencies represent the average of 4–6 independent experiments. Error bars represent the standard deviation. *P*-values are indicated, and were calculated using the Student's *t*-test.

We hypothesized that H_2_O_2_-sensitivity of *dinB* and/or *mutS* strains was the result of their respective inabilities to effectively cope with oxidized DNA lesions. Given that DinB is a TLS Pol, we hypothesized that it might catalyze error-prone bypass of oxidized DNA lesions, resulting in H_2_O_2_-induced mutations. In this case, inactivation of DinB would reduce the frequency of H_2_O_2_-induced mutagenesis. In addition, we hypothesized that independently of DinB, MutS might recognize oxidized DNA lesions, and either directly catalyze their repair, or shuttle them into the appropriate accurate DNA repair pathway. In this case, loss of MutS function would result in accumulation of H_2_O_2_-induced lesions, possibly necessitating their tolerance *via* DinB-mediated TLS. As a test of these hypotheses, we measured H_2_O_2_-induced mutation frequencies for our isogenic *mutS* and/or *dinB* strains. As summarized in Fig. 3A, mutation frequency of the wild-type *P. aeruginosa* strain (MPAO1) was increased ∼10-fold following exposure to H_2_O_2_ (20.7±1.3×10^−8^) compared to the mock treated control (2.2±1.5×10^−8^). Since we propose that DinB activity contributes to this mutator phenotype, we tested if the loss of DinB function impaired H_2_O_2_-induced mutagenesis. Consistent with our hypothesis, the frequency of H_2_O_2_-induced mutagenesis for the *dinB*-deficient strain (5.6±1.0×10^−8^) was ∼4-fold lower than that observed with the wild-type strain (20.7±1.3×10^−8^; see Fig. 3A). Importantly, H_2_O_2_-induced mutagenesis was fully restored by the DinB-expressing plasmid, verifying a direct role for DinB.

We next asked whether MutS function influenced the frequency of H_2_O_2_-induced mutagenesis. As shown in Fig. 3B, the *mutS*-deficient *dinB^+^* strain (MPA32417) displayed a weak H_2_O_2_-induced mutator phenotype that was only ∼1.5-fold higher than its spontaneous mutation frequency (26.5±3.2×10^−6^ for H_2_O_2_-treated cultures compared to 18.2±5.9×10^−6^ for mock-treated controls). Inasmuch as this increase was considerably smaller than the ∼10-fold increase observed for the *mutS^+^ dinB^+^* strain (MPAO1), these results suggest a direct role for MutS in ROS-induced mutagenesis (see *Discussion*). Importantly, H_2_O_2_-induced mutagenesis in the *mutS* strain was still DinB-dependent (Fig. 3B), as we observed a more than 8-fold reduction in the H_2_O_2_-induced mutation frequency for the *mutS dinB* strain (3.3±1.9×10^−6^) compared to the *mutS*-deficient *dinB^+^* strain (26.5±3.2×10^−6^). Consistent with results discussed above, this defect was fully complemented by the DinB-expressing plasmid, providing further support for a role for DinB (Fig. 3B). Remarkably, the frequency of Rif^R^ for the H_2_O_2_-treated *mutS dinB* strain (3.3±1.9×10^−6^) was more than 6-fold lower than the spontaneous mutation frequency observed for the same strain following mock treatment (21.5±4.5×10^−6^), resulting in an overall negative value for H_2_O_2_-induced mutation frequency (−18.2±4.7×10^−6^; Fig. 3B). This difference was reproducibly observed, and was statistically significant (*p*<0.001, based on Student's *t*-test). Taken together, these results support a model in which DinB and MutS act synergistically to cope with oxidized DNA lesions (see *Discussion*).

### *P. aeruginosa* DinB catalyzes bypass of 8-oxo-dG *in vitro*

We interpreted our finding that *dinB* function contributes to H_2_O_2_-induced mutagenesis (Fig. 3) to suggest that DinB catalyzes TLS over one or more classes of oxidized DNA lesions. Since 8-oxo-dG is a well-established ROS-induced lesion [1], we tested whether purified DinB protein could catalyze bypass of template–8-oxo-dG *in vitro*. For these experiments, we utilized a synthetic oligonucleotide substrate comprised of 20-mer template bearing either dG, or 8-oxo-dG positioned immediately downstream of the 3′-OH of an annealed complimentary 13-mer oligo (see legend to Table 3 for oligo sequences). Under single turnover conditions (*e.g*., 200 nM DinB and 100 nM DNA), DinB specifically incorporated dCTP opposite template-dG: we did not detect incorporation of dATP, dGTP, or dTTP (Table 3). In contrast to template-dG, DinB incorporated both dCTP and dATP opposite template–8-oxo-dG: incorporation of dGTP or dTTP was not detected (Table 3). The 2-fold preference for incorporation of dATP compared to dCTP arises primarily through an increase in binding affinity for dATP (K_M_ = 15±5.0 µM) relative to dCTP (K_M_ = 28±7.0 µM; see Table 3). Taken together, these findings indicate that DinB can bypass template–8-oxo-dG in either an accurate manner by inserting dCTP, or an error-prone manner by inserting dATP, contributing to GC→TA transversions. Coincidently, *P. aeruginosa* DinB catalyzes GC→TA transversions when expressed in *E. coli* [20].

**Table 3:** *P. aeruginosa* DinB catalyzes both accurate and error-prone bypass of 8-oxo-dG *in vitro*.^a^

Incorporation of:^b^		Kinetic parameters^c^		
Nucleoside triphosphate	Opposite template base	K_M_ (µM)	k_cat_ (s^−1^)	k_cat_/K_M_ (M^−1^s^−1^)
dCTP	dG	5.4±2.3	0.120±0.010	2.2×10^4^
dCTP	8-oxo-dG	28±7.0	0.066±0.005	0.2×10^4^
dATP	dG	*nd* d	*nd*	*na* e
dATP	8-oxo-dG	15±5.0	0.066±0.006	0.4×10^4^

***^a^***Bypass activity was measured *in vitro* using a synthetic 13-mer oligonucleotide primer (5′-TGG CAG CCG GTC A-3′) annealed to a synthetic 20-mer template strand bearing either dG or 8-oxo-dG (3′-ACC GTC GGC CAG T***x***C CCA AA-5′, where ***x*** represents either dG or 8-oxo-dG).

***^b^***Neither dTTP nor dGTP were incorporated opposite template dG at a detectable level.

***^c^***Values shown represent the average of at least 3 independent experiments, ± the standard deviation.

***^d^***Incorporation of dATP opposite template dG was not detected (*nd*).

***^e^***Not applicable (*na*).

To determine whether GC→TA transversions induced by DinB-mediated bypass of 8-oxo-dG contributes to H_2_O_2_-induced mutagenesis in *P. aeruginosa in vivo*, we analyzed the sequence of the *rpoB* gene from representative Rif^R^ clones (see *Materials and Methods*). As noted above, and summarized in Fig. 1A, the majority of spontaneous mutations resulted from TA→CG transitions, irrespective of *dinB* and/or *mutS* function: 9/12 (75%) Rif^R^ mutations in the wild-type strain resulted from TA→CG transitions, compared to 16/19 (85%) for the *dinB* strain, 18/18 (100%) for the *mutS* strain, and 19/20 (95%) for the *dinB mutS* double mutant. In contrast to spontaneous mutations, which at least in the wild-type strain (MPAO1) were distributed among seven distinct nucleotide positions, corresponding to five different amino acids, H_2_O_2_-treatment resulted in an obvious increase in the frequency of mutation at residue D521, identifying this position as a ‘hot spot’ for ROS-induced Rif^R^ mutations in *P. aeruginosa* (Table 2). Furthermore, with the exception of the Δ*dinB* strain (WFPA334), which contained comparable levels of TA→CG transitions and CG→GC transversions, H_2_O_2_ treatment induced mostly TA→CG transitions (see Fig. 1B & Table 2). It is noteworthy that GC→TA transversions, characteristic of error-prone DinB-mediated bypass of 8-oxo-dG (see Table 3), were not observed. Taken together, these results suggest that *P. aeruginosa* DinB contributes to H_2_O_2_-induced mutagenesis by one or more mechanisms that are functionally distinct from that involving its ability to catalyze error-prone bypass of 8-oxo-dG (see *Discussion*).

### Both DinB and MutS functionality contribute to accurate tolerance of DNA lesions induced by exposure to ultraviolet light (UV), mitomycin C (MMC), or 4-nitroquinilone 1-oxide (4-NQO) *in vivo*

**Figure 4:**
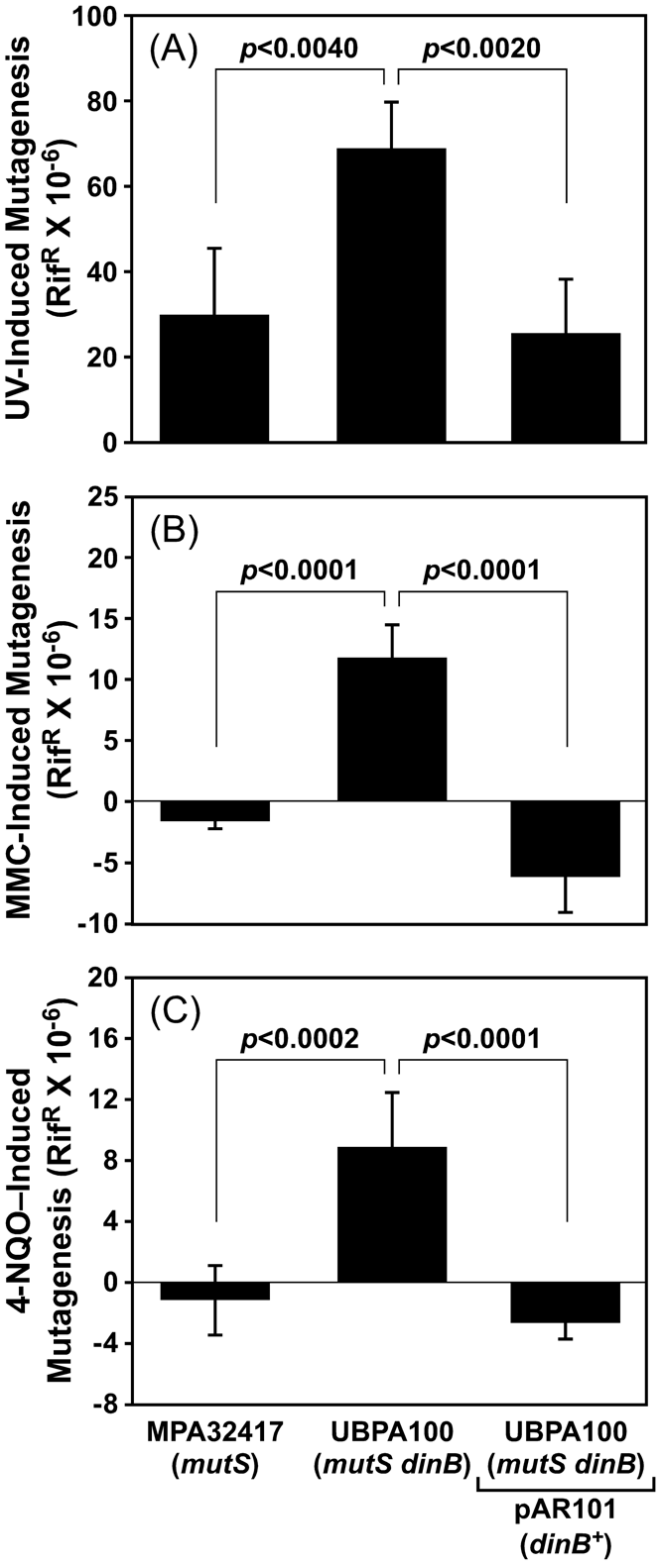
Contribution of *dinB* function to UV-, MMC-, and 4-NQO–induced mutagenesis in *P. aeruginosa*. DNA damage-induced mutation frequencies following exposure of MutS-deficient *dinB^+^* and Δ*dinB*::*aacC1 P. aeruginosa* strains to 25 J/m^2^ of 254 nm UV light delivered from a germicidal bulb (**A**), 1 µg/ml MMC (**B**), or 320 µM 4-NQO (**C**) were determined as described in *Materials and Methods*. Strains examined include MPA32417 (*mutS*::IS*phoA*/hah) bearing pUCP20T, and UBPA100 (Δ*dinB*::*aacC1 mutS*::IS*phoA*/hah) bearing pUCP20T (control) or pAR101 (*dinB^+^*). Induced mutation frequencies represent the average of 5 independent experiments. Error bars represent the standard deviation. *P*-values are indicated, and were calculated using the Student's *t*-test.

As part of an effort to better understand the synergistic relationship between MutS and DinB in H_2_O_2_-induced mutagenesis, we asked whether these proteins played similar roles in tolerating DNA lesions induced by exposure to UV, MMC, or 4-NQO. We first determined contributions of *dinB* and/or *mutS* functionality to UV-induced mutagenesis. Based on results of previously published experiments utilizing *mutS^+^* strains [20], Pol I and PolC are required for the vast majority of UV-induced mutagenesis: in this study, inactivation of *dinB* failed to impact significantly on the frequency of mutations induced by UV irradiation [20]. In order to determine whether MutS (or MMR) function masked an error-prone role for DinB in UV-induced mutagenesis, we analyzed frequencies for *mutS*-deficient *dinB^+^* and Δ*dinB* strains. As summarized in Fig. 4A, the *mutS*-deficient strain (MPA32417) displayed a modest UV-induced mutator phenotype that was similar in magnitude (∼2-fold increase relative to the mock-treated control) to that observed for the isogenic *mutS^+^* strain [20]. These findings indicate that *mutS* functionality does not contribute to UV-induced mutagenesis. In contrast to the *mutS* strain, the *mutS dinB* double mutant (UBPA100) displayed a statistically significant ∼2.3-fold further increase in the frequency of UV-induced mutagenesis (Fig. 4A). This increased frequency of UV-induced mutagenesis was fully complemented by the DinB-expressing plasmid (Fig. 4A), suggesting that DinB is capable of bypassing UV-induced lesions in a relatively accurate manner *in vivo*, and that in its absence, one or more alternative Pols catalyze less accurate bypass of UV-induced lesions (see below). Moreover, these results suggest that MutS plays an accurate role in policing the fidelity of these alternative Pols.

We next examined roles for *dinB* and *mutS* in coping with MMC-induced *N^2^*-dG–*N^2^*-dG interstrand DNA cross-links at 5′-dC–dG-3′ DNA sequences [47]. We previously determined that *P. aeruginosa mutS^+^* strains failed to display an MMC-induced mutator phenotype [20]. Likewise, the *mutS*-deficient strain failed to display a discernable MMC-induced mutator phenotype (Fig. 4B). *E. coli* DinB contributes to the repair of MMC-induced *N^2^*-dG–*N^2^*-dG interstrand DNA cross-links [8]. Consistent with *P. aeruginosa* DinB similarly contributing to repair of MMC-induced lesions, simultaneous inactivation of both *mutS* and *dinB* led to a more than 10-fold increase in the frequency of MMC-induced mutagenesis (Fig. 4B). This robust MMC-induced mutator phenotype was fully complemented by the DinB-expressing plasmid (Fig. 4B). Taken together, these results suggest that DinB contributes to accurate repair/bypass of *N^2^*-dG–*N^2^*-dG interstrand DNA crosslinks, and that in the absence of DinB, another Pol catalyzes error-prone bypass. Moreover, the 10-fold increase in Rif^R^ observed for the *mutS dinB* double mutant suggests that: (i) *P. aeruginosa* has one or more Pol(s) in addition to DinB that is capable of catalyzing bypass of MMC-induced interstrand DNA crosslinks; and (ii) MutS (or MMR) corrects errors catalyzed by the Pol(s) that is used in place of DinB.

The last agent that we examined was 4-NQO. We previously determined that *dinB* function protected *P. aeruginosa* against 4-NQO–mediated killing without significantly affecting mutation frequency, suggesting that DinB catalyzed accurate bypass of 4-NQO–induced DNA lesions [20]. As summarized in Fig. 4C, the *mutS*-deficient strain (MPA32417) failed to display a 4-NQO–induced mutator phenotype. In contrast, the *mutS dinB* double mutant (UBPA100) exhibited a ∼10-fold increase in the frequency of 4-NQO–induced mutagenesis that was fully complemented by a DinB-expressing plasmid (Fig. 4C). Taken together, these results suggest that *P. aeruginosa*: (i) possesses one or more Pols in addition to DinB that contributes to bypass of 4-NQO–induced DNA lesions; and (ii) MutS acts to limit errors catalyzed by the Pol(s) used in place of DinB.

Taken together, results discussed above indicate that DinB function contributes to the accurate tolerance of DNA lesions induced by UV, MMC, or 4-NQO. Importantly, in the absence of DinB, *P. aeruginosa* is able still able to tolerate these lesions, albeit less accurately, possibly through the use of one or more alternative TLS Pols. Importantly, *mutS* function contributes further to the fidelity with which these lesions are tolerated. Finally, these results also demonstrate that the *mutS* strain is capable of a robust DNA damage-induced mutator phenotype (Fig. 4). Thus, the inability of this same strain to display a significant H_2_O_2_-indcued mutator phenotype is not an artifact of the high spontaneous mutation frequency that is characteristic of *mutS*-deficient strains; rather, these results provide further support for a direct role for MutS in promoting H_2_O_2_-induced mutagenesis.

### DinB catalyzes accurate bypass of a *cis-syn* thymine cyclobutane dimer *in vitro*

**Figure 5:**
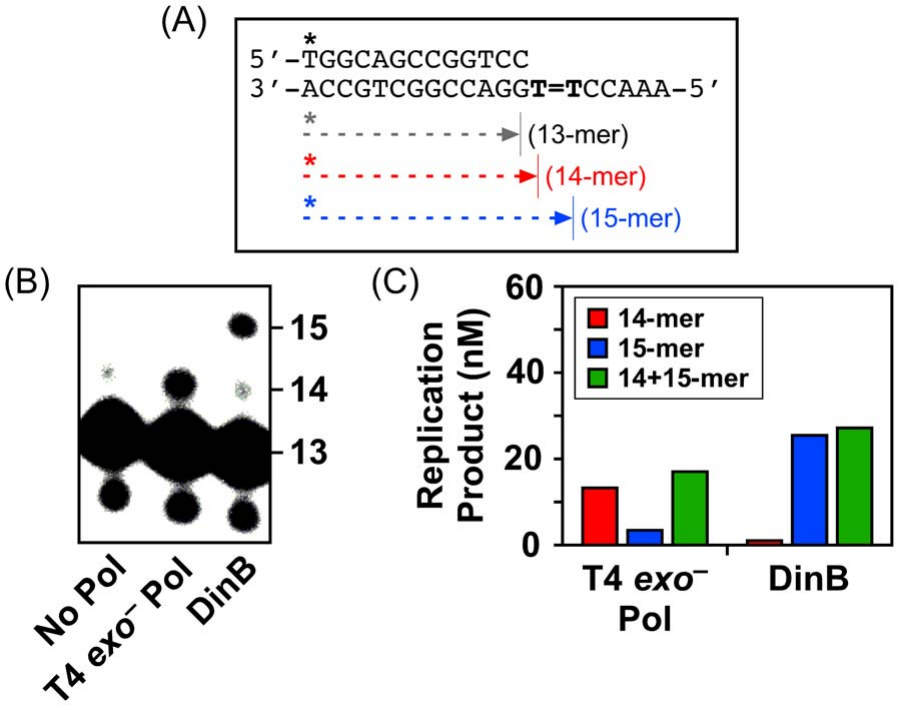
DinB catalyzes accurate bypass of a model *cis-syn* thymine cyclobutane dimer *in vitro*. Cartoon representation of the 13/20_T = T_-mer DNA template (**A**). T = T represents the *cis-syn* thymine cyclobutane dimer. Predicted sizes for the starting primer (13-mer), as well as the different possible bypass products (14-mer & 15-mer) are indicated. Representative bypass results for T4 *exo^–^* Pol and *P. aeruginosa* DinB are shown (**B**). Positions for the primer (13-mer), and the 14-mer and 15-mer bypass products are indicated. Quantitation of the results of a representative bypass assay obtained with both T4 *exo^–^* Pol and DinB are shown (**C**).

**Figure 6:**
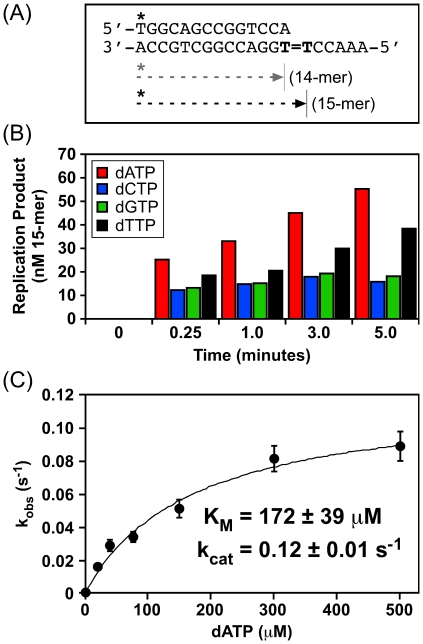
DinB catalyzes accurate bypass of the 5′-dT in a *cis-syn* thymine cyclobutane dimer *in vitro*. Cartoon representation of the 14/20_T = T_-mer DNA template (**A**). T = T represents the *cis-syn* thymine cyclobutane dimer. Sizes for the starting primer (14-mer), as well as the bypass product (15-mer) are indicated. Bypass efficiency of the 5′-dT of the dimer by DinB as a function of time in the presence of each of the four individual dNTPs is shown (**B**). Catalytic efficiency with which DinB mediates bypass of the 5′-dT of the dimer in the presence of dATP (**C**). K_M_ and k_cat_ values shown represent the average of at least 3 independent experiments. Error bars represent the standard deviation.

In contrast to its error-prone role in coping with H_2_O_2_-induced lesions (see Table 3), results summarized in Fig. 4 suggest that DinB catalyzes accurate bypass of DNA lesions induced by UV, MMC, or 4-NQO. In order to obtain biochemical support for this conclusion, we asked if recombinant DinB protein catalyzed accurate bypass of a model *cis-syn* thymine cyclobutane dimer *in vitro*. As a control for these experiments, we utilized an exonuclease proofreading-deficient form of the bacteriophage T4 Pol (T4 *exo^–^* Pol), which incorporates dATP exclusively opposite the 3′-dT of the dimer, yielding almost exclusively a 14-mer product ([48]; see Fig. 5, panels B & C). In contrast to T4 *exo^–^* Pol, DinB catalyzed insertion of dATP opposite both thymines in the dimer, yielding a 15-mer product (∼25 nM), with little to no detectable 14-mer (Fig. 5, panels B & C). This result suggests that incorporation of dATP opposite the 5′-dT of the dimer by DinB is remarkably efficient following bypass of the 3′-dT of the dimer. We therefore investigated the efficiency with which DinB extended a 14-mer primer containing a 3′-dA located opposite the 3′-dT of the dimer (see Fig. 6A). As summarized in Fig. 6 (panels B & C), DinB efficiently extended the 14-mer, preferentially incorporating dATP opposite the 5′-dT of the dimer (K_M_ = 172±39 µM, k_cat_ = 0.12±0.01 s^−1^). These findings, taken together with those discussed above, indicate that DinB bypasses ROS-induced lesions (see Fig. 3 and Table 3) with lower fidelity than a thymine cyclobutane dimer (see Figs. 4A, 5 & 6).

## Discussion

**Figure 7:**
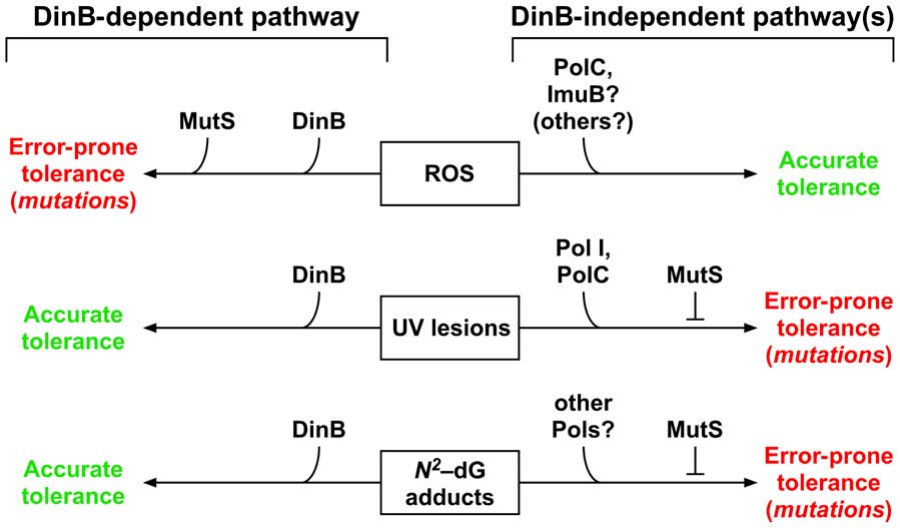
Models to describe roles for *P. aeruginosa* DinB and MutS in DNA repair, DNA damage tolerance, and DNA damage-induced mutagenesis. Proposed roles for MutS, DinB, and additional factors (as noted) in coping with ROS-, UV-, and MMC- or 4-NQO–induced (collectively referred to as ‘*N^2^*-dG adducts’) DNA lesions are summarized. Fidelities (accurate or error-prone) of MutS and DinB in each pathway (DinB-dependent and DinB-independent) are indicated. See text for additional details.

In striking contrast to their largely accurate roles in coping with lesions induced by UV, MMC, or 4-NQO, MutS and DinB activities contribute to H_2_O_2_-induced mutagenesis (Fig. 3). Moreover, in the absence of both DinB and MutS, H_2_O_2_ exposure actually increased the fidelity of *P. aeruginosa* DNA replication by a factor of more than 6-fold as measured by Rif^R^. The increase in replication fidelity without a concomitant increase in the sensitivity of *P. aeruginosa* to H_2_O_2_-induced killing (Fig. 2) suggests the presence of one or more ROS-inducible pathways that are responsible for accurate tolerance of oxidized DNA lesions. A potential candidate is the activity of other Pols. Indeed, *P. aeruginosa polC* and *imuB* encode putative Y-family Pols ([49], [50]; see http://www.pseudomonas.com). Based on results of microarray experiments, transcription of both *polC* and *imuB* is induced following exposure of *P. aeruginosa* to H_2_O_2_ [45], [46]. Thus, these Pols may contribute to accurate bypass of ROS-induced lesions. Alternatively, *polC* and *imuB* function may contribute to error-prone bypass, resulting in the modest level of H_2_O_2_-induced mutagenesis observed in the Δ*dinB* strain (Fig. 7).

Consistent with a role for DinB in contributing to H_2_O_2_-induced mutations by catalyzing error-prone bypass of oxidized DNA bases, we determined that DinB favored incorporation of dATP opposite template–8-oxo-dG over dCTP by a factor of ∼2 *in vitro* (Table 3). However, based on nucleotide sequence analysis of the *rpoB* gene isolated from Rif^R^ mutants, this behavior of DinB does not appear to contribute significantly to H_2_O_2_-induced mutations in *P. aeruginosa* (Table 2 & Fig. 1). Taken together, these results suggest that DinB contributes to mutations by catalyzing error-prone bypass of one or more oxidized DNA lesions other than 8-oxo-dG *in vivo*. Alternatively, *P. aeruginosa* DinB may incorporate oxidized precursors into undamaged DNA. Consistent with this model, *E. coli* DinB catalyzes incorporation of dCTP opposite template–2-hydroxyadenine, 2-hydroxyadenine opposite template-dG (or template-dT), and 8-oxo-dG opposite template-dA. If left unrepaired, these insertions will result in TA→CG, GC→TA or AT→CG substitutions, respectively, following the subsequent round of DNA replication [18], [51]. Nucleotide sequence analysis indicated that although the majority of H_2_O_2_-induced mutations corresponded to TA→CG transitions, this substitution represented less than one-half of the mutations in the Δ*dinB* strain (Fig. 1 and Table 2). These results suggest that DinB-dependent H_2_O_2_-induced mutagenesis may result, at least in part, from error-prone bypass of 2-hydroxyadenine.

An error-prone role for *P. aeruginosa* MutS in coping with oxidized DNA lesions was unexpected. The human MutS ortholog, MutSα, recognizes and binds 2-hydroxyadenine present within poly-dA sequences that mimic an insertion/deletion loop [52]. It is possible that *P. aeruginosa* MutS contributes to ROS-induced mutagenesis by influencing the fidelity with which oxidized lesions are repaired. For example, MutS may play an error-prone role in repairing mismatches catalyzed by DinB, particularly mismatches resulting from bypass of oxidized lesions, and/or incorporation of oxidized precursors (such as 2-hydroxyadenine and 8-oxo-dG), due to an inability to discriminate between the lesion and the undamaged base, resulting in mutations. Alternatively, MutS might recruit DinB to sites of ROS-induced lesions, contributing to their error-prone bypass. Biochemical approaches are required to test these models.

Roles for DinB/Pol κ in catalyzing largely accurate bypass of *N^2^*-dG adducts, such as those induced by 4-NQO and MMC, have been described: *E. coli* DinB and mammalian Pol κ can each bypass *N^2^*-dG furfuryl adducts *in vitro* [6], and *dinB*-deficient *E. coli* and *P. aeruginosa* strains are sensitive to 4-NQO [6], [20]. Furthermore, it was recently reported that both *E. coli* DinB [8] and human Pol κ [9], [53], catalyze bypass of model *N^2^*-dG–*N^2^*-dG interstrand cross-links *in vitro*. Moreover, functionality of these Pols served to protect cells against MMC-induced killing, presumably *via* their involvement in cross-link repair. Finally, DinB and Pol κ also catalyze bypass of DNA-peptide crosslinks involving the *N^2^* position of dG *in vitro* [53]. Taken together, these findings indicate that DinB and Pol κ promote accurate bypass of a variety of different *N^2^*-dG adducts. Our results summarized in Fig. 4B suggest that *P. aeruginosa* DinB plays similar roles. In addition, our findings suggest that *P. aeruginosa* possesses one or more additional Pols that catalyze potentially error-prone bypass of *N^2^*-dG adducts. Our results further suggest that MutS corrects errors made by these alternate Pols, ensuring accurate bypass of *N^2^*-dG adducts *in vivo* (Fig. 7).

Although *E. coli* DinB is unable to bypass UV photoproducts [12], *P. aeruginosa* DinB catalyzes accurate bypass of a *cis-syn* thymine dimer, both *in vivo* (Fig. 4A) and *in vitro* (Figs. 5 & 6). Importantly, our results also indicate that DinB is less efficient at inserting a base opposite the 3′-dT of the dimer, and instead prefers to extend from the 3′-OH of dA paired with the 3′-dT of the dimer (Fig. 6). PolC and Pol I each also contribute significantly to UV-induced mutagenesis in *P. aeruginosa* [20]. Taken together, these findings suggest that DinB may cooperate with one or more of these Pols while tolerating UV adducts. For example, PolC or Pol I may initiate bypass by inserting a nucleotide opposite the 3′-dT of the dimer, which DinB subsequently extends by preferential incorporation of dATP opposite the 5′-dT of the dimer. DinB may then further extend, or it may transfer the 3′-OH end of the nascent DNA strand to PolC or Pol I before ultimately returning it to the replicative Pol (Fig. 7). In the absence of DinB, PolC or Pol I may catalyze error-prone bypass of both the 3′-dT and 5′-dT of the dimer. Finally, MutS may recognize and correct errors catalyzed by PolC and/or Pol I, helping to minimize the frequency of UV-induced mutations.

A significant fraction of *P. aeruginosa* strains isolated from CF airways exhibit a spontaneous hypermutable phenotype due to mutations within *mutS* [24], [25], [28], [42]. As a result of their elevated spontaneous mutation frequencies, these strains are proposed to acquire adaptive mutations more frequently than those that are MMR-proficient [42], [54], [55]. Our results indicate that *mutS*-deficient *P. aeruginosa* strains are hypersensitive to H_2_O_2_ (Fig. 2). In addition, we demonstrate that these strains are impaired for H_2_O_2_-induced mutagenesis (Fig. 3). These results suggest that *mutS*-deficient strains are ill equipped for colonizing and persisting within the highly oxidizing environment of the early CF airways. Although this view is consistent with the finding that *mutS* strains are less efficient than wild-type *P. aeruginosa* at establishing airway infections in mice [28], [43], it is nevertheless inconsistent with the fact that *mutS*-deficient strains are prevalent among the CF population. This dichotomy can be explained by several unique mechanisms. First, it is possible that *mutS* strains colonizing CF airways reside within biofilms, which may help to protect them from ROS-induced cell damage. Alternatively, hypermutability resulting from loss of *mutS* may promote rapid acquisition of compensatory mutations that help to mitigate ROS-sensitivity. Indeed, our finding that the *mutS dinB* double mutant was no more sensitive to H_2_O_2_ than the *mutS* single mutant (Fig. 2), yet displayed a greatly reduced level of H_2_O_2_-induced mutagenesis (Fig. 3), suggests that alternative pathways for coping with ROS-induced DNA damage exist in *P. aeruginosa*. We are currently characterizing MMR-deficient *P. aeruginosa* strains recovered from CF airways to determine whether they exhibit phenotypes comparable to the *mutS* reference strain used in this work.

A recent study by Hogardt and colleagues [25] concluded that *mutS*-deficient *P. aeruginosa* strains co-exist with non-mutator (*e.g*., *mutS^+^*) strains, and predominate the CF airways during end-stage infection. These results suggest that *mutS*-deficient strains may not necessarily have an adaptive advantage over *mutS^+^* strains in all airway niches. Alternatively, it is possible that hypermutability contributes to adaptation of *P. aeruginosa* within certain airway niches, or under specific conditions, such as end-stage infection when airways undergo rapid decline in function. Regardless, these findings in conjunction with our results demonstrating a crucial role for MutS and DinB in coping with ROS-induced DNA damage support an alternative model for pathoadaptation of *P. aeruginosa* colonizing the CF airways. In this model, we propose that the remarkably high levels of ROS generated by chronic CF airway inflammation contribute to excessive DNA damage in *P. aeruginosa*. We hypothesize that this high level of damage acts to saturate accurate DNA repair functions, such as those catalyzed by MutS, due to finite levels of the proteins involved. Saturation of MutS function would hamper repair of ROS-induced lesions, and would also serve to impair MMR, leading to a mutator phenotype. We further hypothesize that as a result of oxidized lesions persisting in the DNA, viability of *P. aeruginosa* becomes dependent upon TLS, catalyzed in large part by DinB (see Fig. 3 & Table 3). As TLS is often error-prone, we expect this to contribute further to the mutator phenotype. One advantage of this model over chronic hypermutability is the fact that the mutator phenotype is reversible. As a result, *P. aeruginosa* can ‘shed’ its mutator phenotype once it has acquired one or more adaptive mutations that serve to protect it from ROS. This ability would minimize the likelihood that members of the *P. aeruginosa* population would accumulate deleterious mutations. At the same time, our postulated saturation-induced mutator phenotype might contribute to mutations in genes encoding proteins that act in DNA repair, such as *mutS*, resulting in hypermutable *P. aeruginosa* strains. Although our model postulates that the induced mutator phenotype contributes to adaptation, it is possible that chronic hypermutability *per se* does not. In this case, hypermutators may persist by hitchhiking along with truly adaptive mutations, as discussed previously [56]. In conclusion, irrespective of whether an increased mutation frequency is strictly required for *P. aeruginosa* pathoadaptation in CF, the ability of this pathogen to display a reversible mutator phenotype likely contributes to airway colonization, persistence, and pathoadaptation, particularly under conditions of ROS-induced stress.

ROS-sensitivity of the *mutS* and/or *dinB* strains is reminiscent of phenotypes described for *P. aeruginosa* strains impaired for function of the ‘GO’ component of base excision repair, due to mutations within *mutM*, *mutY*, or *mutT*, which act independently to limit GC→TA transversions resulting from 8-oxo-dG, as well as other oxidized DNA lesions [57], [58]. Although *P. aeruginosa* strains deficient for ‘GO’ function display a level of H_2_O_2_ sensitivity that is similar to that observed for the *mutS* strain, they exhibit significantly increased levels of H_2_O_2_-induced mutagenesis, consistent with ‘GO’ function acting to limit ROS-induced mutations [58]. Of relevance to our model discussed above, *E. coli* MutS is suggested to contribute to MutY function *in vivo* [37]. Thus, under our model, saturation of MutS impacts not only on error-prone tolerance of ROS-induced lesions and MMR, but may impact on ‘GO’-catalyzed repair of ROS-induced lesions, as well, perhaps leading to an even greater spectrum of mutations that may contribute to pathoadaptation of *P. aeruginosa*. Collectively, these findings illustrate the complexity of the phenotypes that may be observed with hypermutable *P. aeruginosa* strains, and further highlight the importance of carefully analyzing these strains under a variety of CF-relevant conditions as part of a comprehensive effort aimed at defining the contribution of mutagenesis to *P. aeruginosa* virulence and pathoadaptation.

In summary, findings discussed in this report reveal previously unrecognized roles for MutS and DinB, and additionally uncover a functional relationship between these proteins in modulating the mutation frequency of *P. aeruginosa* following exposure to ROS. Roles for MutS and DinB in ROS-induced mutagenesis are particularly fascinating given the significance of *P. aeruginosa* to CF airway disease. Continued genetic and biochemical characterization of MutS, DinB, as well as additional factors involved in *P. aeruginosa* MMR, TLS, and DNA repair will expand our understanding of these important metabolic pathways, and should provide crucial insights into their contribution(s) to pathoadaptation of this important human pathogen.

## Materials and Methods

### Commercial reagents and recombinant proteins

Unlabeled ultrapure dNTPs were obtained from GE Healthcare. [γ-^32^P]–ATP was purchased from M.P. BioMedicals (Irvine, CA). Chemicals were obtained from Sigma-Aldrich. Oligonucleotides used for PCR or nucleotide sequence analysis were synthesized by Sigma-Aldrich. The oligonucleotide containing a *cis-syn* thymine cyclobutane dimer (20_T = T_-mer; see panel A, Figs. 6 & 7) was synthesized by TriLink Biotechnologies (San Diego, CA). All other oligonucleotides utilized for enzymatic assays, including that containing 8-oxo-dG (20_8-oxo-dG_-mer; see legend to Table 3), were synthesized by Operon Technologies (Alameda, CA). Single strand DNA was purified by denaturing gel electrophoresis and quantified as described [59]. Duplex DNA was prepared by annealing stoichiometric quantities of the primer strand oligonucleotide with the template strand oligonucleotide, and subsequent purification of the duplex template by non-denaturing gel electrophoresis as described [59]. The C-terminally hexahistidine-tagged form of the *P. aeruginosa* DinB protein [20], and the exonuclease-deficient (D219A) bacteriophage T4 DNA polymerase (T4 *exo^–^* Pol) mutant were purified and quantified as described [60], [61].

### Bacteriological techniques

*P. aeruginosa* strains used in this study are derived from PAO1, and the salient features of each are detailed in Table 4. Strains were routinely grown in Luria-Bertani (LB; 10 g/l tryptone, 5 g/l yeast extract, 10 g/l NaCl) medium [62], unless stated otherwise. When necessary, the following antibiotics were used at the indicated concentrations: rifampicin (Rif), 100 µg/ml; gentamicin (Gent), 100 µg/ml; carbenicillin (Carb), 250 µg/ml; and tetracycline (Tet), 60 µg/ml.

**Table 4:** *P. aeruginosa* strains and plasmid DNAs used in this study.

*P. aeruginosa* strains^a^		
Strain	Relevant genotype	Source or construction
MPAO1	Prototroph	[63]
WFPA334	Δ*dinB*::*aacC1* (Gent^R^)	[20]
MPA32417	*mutS*::IS*phoA*/hah	[63]
UBPA100	*mutS*::IS*phoA*/hah Δ*dinB*::*aacC1* (Gent^R^)	This work

Plasmid DNAs^a^		
Plasmid	Relevant characteristics	Source or construction
pEX18Ap	Ap^R^ (Carb^R^); suicide replacement vector containing *B. subtilis sacB* gene	[65]
pLS100	Ap^R^ (Carb^R^), Gent^R^; pEX18Ap bearing *P. aeruginosa* Δ*dinB*::*aacC1* (Gent^R^) allele	This work
pUCP20T	Ap^R^ (Carb^R^); shuttle vector that replicates in both *P. aeruginosa* and *E. coli*	[69]
pAR101	Ap^R^ (Carb^R^); pUCP20T bearing *P. aeruginosa dinB^+^*	[20]
p*mutS*	Ap^R^ (Carb^R^); pUCP20T bearing *P. aeruginosa mutS^+^*	This work

***^a^***See *Materials and Methods* for details regarding construction of *P. aeruginosa* strains and plasmids DNAs.

*P. aeruginosa* strains MPAO1 (prototroph; identical to PAO1) and MPA32417 (*mutS*::IS*phoA*/hah) were obtained from the University of Washington Genome Center [63]. MPA32417 contains an IS*phoA*/hah transposon inserted after nucleotide 361 (amino acid 121) of the *mutS* coding sequence (the *mutS* ORF consists of 2,568 bases, encoding 856 amino acids). The *mutS*::IS*phoA*/hah allele in MPA32417 was verified by diagnostic PCR using a protocol provided by Dr. Michael Jacobs of the University of Washington Genome Center. Briefly, primers homologous to either the 5′- (PAmutSF; 5′-CAG GCA CAT ATG ACC GAC CTC TCC CAG CAC-3′) or the 3′-end (PAmutSR; 5′-GAG TGT GGA TCC TCA GAC CCG CAT CTT CC-3′) of the *mutS* gene were paired with a primer homologous to the transposon (hah minus 138; 5′-CGG GTG CAG TAA TAT CGC CCT-3′). No product was detected using the PAmutSF-hah minus 138 pair, while the expected ∼2,300 bp product was observed using the PAmutSR-hah minus 138 pair, verifying both the presence and the correct orientation of the transposon insertion in *mutS*. Finally, primers PAmutSF and PAmutSR were used to verify the absence of an intact copy of *mutS*.

*P. aeruginosa* strain UBPA100 (*mutS*::IS*phoA*/hah Δ*dinB*::*aacC1*) was constructed using the protocol described by Schweizer and colleagues [64]. Briefly, plasmid pLS100 was electroporated into strain MPA32417 (relevant genotype: *mutS*::IS*phoA*/hah). Colonies were screened for Gent^R^ and sucrose resistance as described previously [65], [66]. The presence of the Δ*dinB*::*aacC1* allele was confirmed by PCR analysis using primers PAgent59F (5′-GAG ATG CGC GAC GAC-3′) and PAgent31R (5′-CTG CAG GTC GAG CAG G-3′), which flank the *aacC1* cassette located within the *dinB* gene to amplify an ∼2,000-bp fragment consisting of the Gent cassette and flanking *dinB* sequence. In addition, primers PAdinBSalIF (5′- GGT TCG TCG CCG AGT TG-3′) and PAdinBSalIR (5′-CAC CGG TCG CTC GTC GAT AC-3′) were used to amplify the region of *dinB* replaced with the *aacC1* cassette to verify the absence of a wild-type copy of the *dinB* allele.

Plasmid DNAs used in this study are described in Table 4. Plasmid pLS100 was constructed by sub-cloning the PCR amplified Δ*dinB*::*aacC1* cassette from strain WFPA334 into pEX18Ap [20], [65]. The *mutS* gene, complete with its native promoter, was PCR amplified from *P. aeruginosa* MPAO1 genomic DNA using primers PAmutSPROMF (5′-GAT CAG GAG CTC GGT CTA CGC GAC AGG AG-3′) and PAmutSKpn1R (5′-GCT CAT GGT ACC TCA GAC CCG CAT CTT C-3′). The amplified DNA fragment was blunt-end cloned into the pCR-Blunt II-TOPO vector (Invitrogen). The *SacI*-*KpnI* fragment containing *mutS* from this TOPO construct was sub-cloned into plasmid pUCP20T, resulting in plasmid p*mutS*.

### Determination of spontaneous mutation frequency

*P. aeruginosa* cultures were inoculated from single colonies and grown for 16 hrs in 5 ml of LB containing the appropriate antibiotics. Appropriate dilutions of each culture were plated onto LB plates to determine the number of viable cells, and LB plates supplemented with 100 µg/ml of Rif to identify spontaneous *rpoB* mutants. Spontaneous mutation frequency was calculated by dividing the number of Rif^R^ cells by the total number of viable cells in the culture.

### Determination of H_2_O_2_ sensitivity

Cultures of the indicated *P. aeruginosa* strains were grown in LB medium containing the appropriate antibiotics to an OD_595_ of ∼0.5. Cells from 1 ml of culture were collected by centrifugation, resuspended in 1.0 ml sterile 0.8% NaCl, and immediately treated with the indicated concentration of H_2_O_2_ (Sigma-Aldrich; 9.8 M stock). After 15 min, H_2_O_2_ was removed by washing the cells with 1 ml of sterile 0.8% NaCl. H_2_O_2_-induced killing was measured by immediately spreading appropriate dilutions of washed cells onto LB plates, followed by incubation overnight at 37°C. Survival of each strain was calculated relative to a mock-treated control.

### Determination of H_2_O_2_-induced mutation frequency

H_2_O_2_-induced mutagenesis was measured following exposure to 25 mM H_2_O_2_, as described previously [58]. Following treatment as described above, 4.5 ml of fresh LB containing the appropriate antibiotics was inoculated with 0.5 ml of washed cells, followed by growth at 37°C until saturated. Appropriate dilutions of each culture were plated onto LB plates with or without Rif. Mutation frequency was calculated by dividing the total number of Rif^R^ clones present in 1.0 ml of culture by the total number of viable cells in that same culture. H_2_O_2_-induced mutation frequency was calculated by subtracting the spontaneous frequency of Rif^R^ from that observed following H_2_O_2_-treatment.

### Determination of catalase activity in cell-free extracts

Cell-free extracts were prepared from sonicated stationary-phase cells suspended in 50 mM potassium phosphate buffer (pH 7.0; KPi). Catalase activity was measured in triplicate by monitoring the decomposition of 19.5 mM H_2_O_2_ in KPi at 240 nm. One unit of catalase activity is defined as that which decomposes 1 mmol of H_2_O_2_ min^-1^ mg protein^-1^. Protein concentrations were estimated by the method of Bradford [67] using bovine serum albumin fraction V (Sigma-Aldrich) as a standard.

### DinB-mediated bypass of 8-oxo-dG and *cis-syn* thymine cyclobutane dimer *in vitro*

Kinetic rate and Michaelis-Menten constants for dNTP incorporation opposite 8-oxo-dG or a *cis-syn* thymine cyclobutane dimer were determined using 100 nM 13/20_8-oxo-dG_-mer or 250 nM 13/20_T = T_-mer (or 14/20_T = T_-mer, as noted), respectively, and variable concentrations of nucleotide (5-500 µM). Components were pre-incubated in assay buffer (25 mM Tris-OAc [pH 7.5], 150 mM KOAc, 10 mM 2-mercaptoethanol), then mixed with 200 nM DinB (or T4 *exo^–^* Pol) and 10 mM MgCl_2_ at 25°C. Reactions were quenched with 500 mM EDTA at the indicated times (5–300 s). Polymerization was monitored by analysis of products on 20% sequencing gels [59]. Gel images were captured using a Packard PhosphorImager equipped with OptiQuant software. Product levels were determined by measuring the ratio of ^32^P-labeled extended and non-extended primer after subtracting background levels for product levels observed in the absence of added Pol (zero point). Corrected ratios were multiplied by the concentration of primer/template used in each assay to determine total product yield. Data obtained for single turnover DNA polymerization assays were fit to *equation 1*:



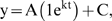
(1)where A is the burst amplitude, k is the observed rate constant (k_obs_) in initial product formation, t is time, and C is a defined constant [59]. Data for the dependency of k_obs_ as a function of dNTP concentration were fit to the Michaelis-Menten equation (*equation 2*) to provide values corresponding to k_cat_ and K_M_:



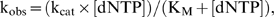
(2)where k_obs_ is the observed rate constant of the reaction, k_cat_ is the maximal polymerization rate constant, K_M_ is the Michaelis-Menten constant for dNTP, and [dNTP] is the concentration of nucleotide substrate [60].

### Nucleotide sequence analysis of the *P. aeruginosa rpoB* allele from Rif^R^ clones

Rif^R^ *P. aeruginosa* colonies were selected for each strain examined as described above. Eighteen-to-twenty independent colonies for each strain (for spontaneous as well as H_2_O_2_-induced) were grown overnight in LB broth supplemented with Rif. A 250-base pair fragment of the *P. aeruginosa rpoB* gene corresponding to amino acid residues 499-582 was PCR amplified from 2 µl of each culture using primer A1 (5′-GCC TCC CTC GCG CCA TCA GAG CGC GCC AAG CCG GTG GCT GCC-3′) and primer B1 (5′-GCC TTG CCA GCC CGC TCA GGG TCG CCA GGG AGT TGA TCA GAC C-3′) as described previously [58]. PCR products were purified using the Qiaprep Mini-spin kit (Qiagen) as per the manufacturer's recommendations. The nucleotide sequence of each purified PCR product was determined by the Biopolymers Facility at Roswell Park Cancer Center (Buffalo, NY) using primer A1.

### Determination of ultraviolet light (UV), mitomycin C (MMC), or 4-nitroquinilone 1-oxide (4-NQO) -induced mutation frequency

UV-, MMC-, and 4-NQO–induced mutation frequencies were measured as described previously [20]. Values reported represent the frequency of Rif^R^ observed following exposure to UV (25 J/m^2^), MMC (1 µg/ml), or 4-NQO (320 µM) after subtracting the spontaneous Rif^R^ frequency for the same strain.
